# Linear Growth in Children and Adolescents with Type 1 Diabetes Mellitus

**DOI:** 10.3390/ijerph16193677

**Published:** 2019-09-30

**Authors:** Elisa Santi, Giorgia Tascini, Giada Toni, Maria Giulia Berioli, Susanna Esposito

**Affiliations:** 1 Pediatric Clinic, Department of Surgical and Biomedical Sciences, Università degli Studi di Perugia, 06123 Perugia, Italy; elisa.santi1988@gmail.com (E.S.); giorgia.tascini@gmail.com (G.T.); toni.giada@gmail.com (G.T.); mgiuliaberioli@gmail.com (M.G.B.); 2 Pietro Barilla Children’s Hospital, Department of Medicine and Surgery, University of Parma, 43126 Parma, Italy

**Keywords:** diabetes mellitus, GH, IGF-1, linear growth, type 1 diabetes

## Abstract

Ensuring normal linear growth is one of the major therapeutic aims in the management of type one diabetes mellitus (T1DM) in children and adolescents. Many studies in the literature have shown that pediatric patients with T1DM frequently present some abnormalities in their growth hormone (GH)/insulin-like growth factor-1 (IGF-1) axis compared to their healthy peers. Data on the growth of T1DM children and adolescents are still discordant: Some studies have reported that T1DM populations, especially those whose diabetes began in early childhood, are taller than healthy pediatric populations at diagnosis, while other studies have not found any difference. Moreover, many reports have highlighted a growth impairment in T1DM patients of prepubertal and pubertal age, and this impairment seems to be influenced by suboptimal glycemic control and disease duration. However, the most recent data showed that children treated with modern intensive insulin therapies reach a normal final adult height. This narrative review aims to provide current knowledge regarding linear growth in children and adolescents with T1DM. Currently, the choice of the most appropriate therapeutic regimen to achieve a good insulin level and the best metabolic control for each patient, together with the regular measurement of growth parameters, remains the most important available tool for a pediatric diabetologist. Nevertheless, since new technologies are the therapy of choice in young children, especially those of pre-school age, it would be of great interest to evaluate their effects on the growth pattern of children with T1DM.

## 1. Introduction

Type 1 diabetes mellitus (T1DM) is the most common chronic metabolic disorder in children. However, the incidence of childhood onset T1D varies significantly between and within countries, ranging from 0.1 cases in Venezuela to more than 40 cases in Finland per 100,000/year [1] In recent years, it has been calculated that T1DM is increasing at a rate of ~3–5% per year. TIDM is an autoimmune disease that is characterized by slowly progressing pancreatic beta-cell destruction, resulting in a reduction of insulin secretion and, over the course of several years, the development of clinically evident insulin-dependent diabetes mellitus [1]. The involvement of the immune system in disease development has been known for many years, as evidenced by the detection of antibodies that recognize pancreatic beta-cell components and insulin in the blood of individuals several years before T1DM development.

Linear growth in children is a complex physiological process influenced by many endocrinological, nutritional, and psychological factors. The effects of T1DM on growth are still unclear and debated in the literature. Early studies showed that chronic hyperglycemia impaired linear growth. Mauriac syndrome [1,2], characterized by growth failure and hepatomegaly, represents the most severe growth-related condition linked to long-term, poorly controlled T1DM with severe insulin deficiency. Currently, Mauriac syndrome is a very rare condition, and the prognosis of growth has improved due to new insulin treatment regimens and systematic glycemic control by patients. The aim of this narrative review is to summarize the current knowledge regarding linear growth in children and adolescents with T1DM. The MEDLINE and PubMed databases were searched for all of the studies published over the last 15 years using the key words “type 1 diabetes” AND “growth” or “height.” Only articles published in English were considered and discussed

## 2. The GH/IGF1 Axis in Children with Type 1 Diabetes Mellitus (T1DM)

Growth hormone (GH) is one of the most important regulatory factors for longitudinal bone growth in children and adolescents [3,4]. Most of the physiological actions of GH are explained by insulin-like growth factor-1 (IGF-1) and insulin-like growth factor-2 (IGF-2) peptides. IGF-1 is reversibly associated with specific insulin-like growth factor binding proteins (IGFBPs). To date, six different IGFBPs have been sequenced [5], and each of them plays a different role, including IGF transport, the modulation of IGF interactions with cell receptors, and the clearance of IGFs [6]. IGFBP-3, whose production is enhanced by GH, is the predominant circulating binding protein and prevents interactions between IGF-1 and its receptors by binding IGF-1 in a ternary complex with an acid-labile subunit [6]. IGFBP-1 is a negative regulator of IGF1 bioactivity, and its hepatic production is inhibited by insulin [7,8,9,10]. Insulin has several effects on the GH/IGF-1 axis: It modulates the liver expression of the GH receptor (GHR) and interacts with GH post-receptor signaling, thus regulating the hepatic synthesis of IGF-1 and IGFBPs [8,11,12,13,14].

It has been demonstrated that, especially during puberty, patients with T1DM present some alterations in the GH/IGF-1 axis, characterized by GH hypersecretion, a reduction in IGF-1 and IGFBP-3 serum, and an increase in IGFPB-1 levels [8,15,16,17,18,19,20,21,22,23,24,25]. Reduced IGF-1 serum levels seem to be due to low intraportal insulin concentration: In fact, exogenous subcutaneous insulin therapy is not able to replace pancreatic insulin secretion in portal circulation [14,26]. These anomalies have been described in particular in children and adolescents with T1DM and poor metabolic control [21,27,28,29,30,31,32,33,34,35,36]. Several studies have found the lowest IGF-1 levels in children with the highest HbA1c levels [21,35,36]. Moreover, other studies have demonstrated an increase in circulating IGF-1 levels after the improvement of intensive insulin therapy [37,38,39]. Some authors have hypothesized that an increased inflammatory response, with an elevated production of interleukin (IL)-8 and IL-6, may be a cause of reduced levels of IGF-1 [22]. Van Sickle et al. conducted a study on 36 adolescents with T1DM and found that the group with higher HbA1c values presented higher levels of IL-8 and lower IGF-1 serum concentrations than the group with better metabolic control [40].

It is noteworthy that in T1DM patients, low IGF-1 levels cause reduced negative feedback to the pituitary gland and, consequently, the hypersecretion of GH [23,41,42]. High GH serum concentration is one of the most important factors contributing to the insulin resistance that is typical of T1DM during pubertal age [20,43,44]. Furthermore, an insufficient intraportal insulin concentration leads to increased production of IGFBP-1, which inhibits IGF-1 bioactivity [20,21,24,45].

## 3. Height at Diagnosis of Type 1 Diabetes Mellitus (T1DM) and Growth Before Puberty

Height at the diagnosis of T1DM in children is a debated topic in the literature. Many studies have reported that children at the onset of T1DM were taller than their healthy peers [27,46,47,48,49,50,51,52,53,54,55,56,57,58,59], while other authors have not confirmed these data [29,30,60,61,62] (Table 1). A possible reason for the differences between these results could be the secular trend in growth (i.e., the fact that puberty is starting earlier for boys and girls now than it did a hundred years ago) and an inadequate choice of controls [63].

Brown et al., along with other authors [28,64], demonstrated an increased height at T1DM onset in children diagnosed between five and 10 years of age compared with healthy peers, but they did not find any significant differences in patients with earlier or later onset [63]. A recent study by Bonfing et al. that analyzed 22,651 German and Austrian children with T1DM showed that their mean height standard deviation (SDS) at diagnosis was significantly higher than the average for the healthy population [65].

Based on these observations, many investigators have studied the growth pattern in children before T1DM onset as a risk factor for the development of the disease. The EURODIAB Study Group conducted a retrospective study showing that height standard deviation (SDS), weight SDS, and body mass index (BMI) SDS were significantly increased in T1DM patients compared to healthy children, with the maximum differences occurring between one and two years of age, thus demonstrating that increased early growth could be associated with disease risk in European populations [66]. Moreover, many recent studies have confirmed that a rapid height, weight and BMI gain in early childhood seems to be linked to the development of islet autoimmunity and to the subsequent onset of T1DM in children [67,68,69,70,71,72,73].

In contrast, Bizzarri et al. evaluated 104 children of prepubertal age and confirmed their increased height at T1DM diagnosis but did not find any correlation between height (or BMI) and age at disease onset [54]. As a possible mechanism to explain the increased height of children at T1DM onset, the authors suggest augmented IGFBP-3 proteolysis, with a consequent increase in IGF-I availability, due to the progressive insulinopenia during the prediabetic period. In the same study, the authors showed that metabolic control (expressed as HbA1c levels) influenced the growth pattern and demonstrated that height velocity after T1DM diagnosis was directly correlated with pancreatic beta cell residual activity, evaluated as C-peptide levels [54]. Several reports have shown that children with T1DM present a reduction in height SDS and growth velocity after disease onset during the prepubertal age [27,51,54,56,57,64,74,75]. Furthermore, the growth rate seems to be influenced by age at T1DM onset, with a the most severe growth impairment occurring in children with disease diagnosis in early childhood [31,57,65,74].

## 4. Growth during Puberty and Final Adult Height in Patients with Type 1 Diabetes Mellitus (T1DM)

Puberty is a fundamental event in human growth that leads to the acquisition of final adult height and to the attainment of reproductive capacity. During puberty, there is a notable increase in growth velocity (the pubertal growth spurt) that is regulated by a subtle interaction of GH/IGF-1 and gonadotropin releasing hormone (GnRH)/luteinizing hormone (LH)-follicle stimulating hormone (FSH)/sex steroid axes [76]. Both estrogens and androgens have important effects on linear growth and increase the amplitude of pulsatile GH secretion, with a physiological rise in GH and IGF-1 levels during puberty [31,41,77]. However, there are some substantial sex-dependent differences in growth pattern, puberty duration and height gain: It is well known that boys have a higher growth velocity peak during mid-puberty, while girls have the a greater height velocity at the beginning of puberty.

During pubertal age, increased GH secretion and the consequent increase in insulin resistance, together with concomitant behavioral factors, exacerbate the abnormality of metabolism in most adolescents with T1DM [78]. Many studies have reported a reduction in height velocity during puberty [30,46,52,58,63,64,79,80,81] in adolescents with T1DM, and this seems to be linked to their reduced IGF1 serum level compared to healthy peers [16,31]. While it is well known that the pubertal growth spurt of patients with T1DM has a normal onset age and an unchanged duration [16,82], it is debated in the literature whether there are any sex-specific differences in height growth velocity. Du Caju et al. [30] showed an impaired pubertal spurt only in the female study group, with a final stature lower than their parental target height. Similar results were reported by other authors in the literature [16,46,63,79,80,83]. In contrast, Kanumakala et al [84], in their analysis of 99 T1DM children, reported impaired linear growth during puberty only in boys.

Plumper et al. analyzed data from 1294 patients with T1DM, ranging in age from seven to 16 years, and demonstrated other important sex-specific differences: They found a significant reduction in the mean maximal growth velocity by 1.2 cm/year only in boys during the pubertal growth spurt, while girls, after reaching peak of height velocity, presented a pattern characterized by a faster decline in growth velocity than the reference population [82]. Moreover, Bizzarri et al. showed a higher serum level of IGF-I and a higher height velocity in adolescent girls than in boys, and the differences did not depend on metabolic control (height velocity SDS: Females = 0.6 ± 2.4 vs. males = −0.45 ± 2.3 cm, P= 0.04; IGF-I SDS: Females = −1.09 ± 0.58 vs. males = −1.4 ± 0.6 cm, *p* = 0.02); to explain these differences, they hypothesized that estrogens may have a stronger impact than androgens on GH signaling [31].

Though some reports did not show the influence of metabolic control on pubertal growth in T1DM patients [27,79,84,85,86], many studies have demonstrated that high HbA1c levels and poor glycemic control are correlated with reduced height growth in adolescents with T1DM [16,31,57,58,81,82,87,88,89,90]. Marcovecchio et al., in a recent study on 206 adolescents with T1DM, showed a significant impairment in pubertal growth with a height SDS reduction from 0.145 to −0.003 cm (*p* < 0.001) and a worse growth trend in adolescents who subsequently developed microalbuminuria, who also had worse metabolic control than those who did not develop microalbuminuria [81].

Despite the growth anomalies described, most studies have shown that children with T1DM reach a normal or only slightly reduced final height compared to the reference population [30,32,46,51,56,59,74,83,91] (Table 1). In agreement with these findings, Brown et al., in their study on 184 children with T1DM, reported that, despite a reduced growth spurt during puberty, the children attained a final height corresponding to the mid-parental height. However, some studies have demonstrated a correlation between T1DM duration and final adult height, with worse growth in patients with a longer duration of diabetes [57,58,63,65,90].

Metabolic control is another factor that influences final height in T1DM patients [57,92,93,94]. Bonfig et al. collected data on adult height from 1685 German children and found a mean final adult height of 167.1 ± 6.2 cm (−0.16 ± 0.97 SDS) in females and 179.6 ± 7.1 cm (−0.17 ± 1 SDS) in males, without any significant differences between sexes; in the same study, when the subjects were divided into different groups based on glycemic control, they found that the group with HbA1c <7.0% had a final adult height SDS of + 0.030 cm, while the groups with HbA1c 7.0–8.0% and suboptimal metabolic control (HbA1c >8.0%) had a final adult height SDS of −0.122 and −0.308 cm, respectively [65].

**Table 1:** Main studies on linear growth parameters in children and adolescents with type 1 diabetes mellitus (T1DM).

Study	Study Population Number Age (Range) years	H at T1DM onset (Mean SDS ± SD)	Final H (Mean SDS ± SD)	Other	Conclusions	
**Cianfarani 2000** [29]	60 (35 M, 25 F) (2.5–10) yrs	cases 0.64 ± 1.4 vs. controls 0.64 ± 0.9*; P ns	Nd		H at T1DM onset was not significantly different from H of healthy peers.	Increased IGFBP-3 proteolytic activity and reduced IGF-1 levels in T1DM children.
**Bizzarri 2014** [31]	91 (54 M, 37 F) (4.1–15.3) yrs	Nd	Nd	HV during puberty (mean SDS ± SD): M: −0.45 ± 2.3 vs. F: 0.6 ± 2.4; *p* = 0.04	HV SDS was higher in F than in M during pubertal age.	IGF-I SDS and IGF-I/IGFBP-3 molar ratio were significantly lower in M than in F. No differences in CSII or MDI therapy.
**Chiarelli 2004** [32]	30 (30 M) Prepubertal: 10.3 ± 2.0 yrs Pubertal: 13.8 ± 1.1 yrs Post-pubertal: 18.5 ± 1.3 yrs	Nd	Nd	H (SDS ± SD): Prepubertal: 0.68 ± 0.9 vs. 0.46 ± 0.9*, P ns Pubertal: 0.15 ± 1.6 vs. 0.17 ± 1.33*, P ns Post-pubertal: 0.19 ± 0.8 vs. 0.18 ± 0.7* P ns	H SDS was not different from that of healthy peers.	No statistically significant differences in IGF-1 and IGFBP3 serum concentrations between T1DM groups and control subjects.
**Mao 2011** [53]	57 (26 M, 31 F) (6–10) yrs	M: 0.34 ± 0.93 F: 0.38 ± 0.50	M: –0.42 ± 0.95 F: –0.60 ± 0.98	H at the start of puberty: M: –0.66 ± 1.07 F: –0.93 ± 1.13	H at diagnosis was higher than healthy peers in both sexes. Final H was reduced compared to the healthy group.	
**Bizzarri 2013** [54]	104 (51 M, 53 F) (1.02–11.08) yrs	0.52 ± 1.04	H at puberty onset: 0.36 ± 1.10	HV: –0.14±1.84^ ΔH: –0.15±0.83^	Increased H at onset in prepubertal children in comparison with reference standards.	HV was directly affected by C-peptide (*p* = 0.03) and insulin requirement (*p* = 0.004) and inversely related to HbA1c (*p* = 0.006).
**Lebl 2003** [56]	587 (317 M, 270 F) (0.3–20.3) yrs	F: 0.74 ± 1.46 M: 0.15 ± 1.1	Mean final H (cm) (approximately 123 patients): F 167 (GTH 165.6) M 176.5 (GTH 176.8)		H at diagnosis was significantly greater than standards population both in M and in F. Significant lose of H after diagnosis in M (*p* < 0.001).	HbA1c did not correlate with growth pattern.
**Elamin 2006** [58]	72 (37 M, 35 F) (7–13) yrs	M: 0.04 ± 0.96 F: 0.15 ± 0.40	M: −1.63 ± 0.44 F: -1.72 ± 0.79	Pubertal H gain ΔH cm (range) M: 20.6 (13.0 – 28.0) F: 17.9 (12.0 – 23.0)	H at diagnosis was greater than GTH. Significant reduction in pubertal growth spurt. Mean final H attained was lower than GTH.	
**Bizzarri 2018** [59]	104 (51 M, 53 F) (5.91 ± 1.9) yrs	Mean H SDS (±SD) 0.52 ± 1.04 vs GTH SDS 0.01 ± 1.02 *p* < 0.05	M 0.04 ± 1.1 F –0.27 ± 1.0 P ns	Pubertal H gain ΔH cm (range) M 24.4 ± 4.9 cm F 19.0 ± 3.8 cm	H at the onset was significantly higher than GTH. H at puberty onset and final H were not different from GTH.	No statistically significant differences in growth between patients treated with CSII and MDI. Final H was positively related to the daily dose of basal insulin during puberty.
**Bonfig 2012** [65]	1685 (839 M, 846 F) <11 yrs	All: 0.22 ± 1	M: −0.17 ± 1 F: −0.16 ± 0.97		H at onset is above mean standard values. Final H was normal in patients with a mean HbA1c <7.0%, but significantly decreased in those with mean HbA1c 7.0%-8.0% and mean HbA1c >8.0%.	Final H was positively correlated with H at onset (*p* < 0.0001) and negatively correlated with mean HbA1c (*p* < 0.0001) and duration of diabetes (*p* = 0.0015).
**Wong 2000** [79]	58 (22 M, 36 F) (1.75–14.79) yrs	M: + 0.76 vs. F: −0.07 *p* = 0.015	M + 0.14 F - 0.57		H at diagnosis in M was significantly greater than F and than standards population (*p* = 0.015); Mean final H in F was shorter than their GTH ( *p* = 0.04) while was normal in M.	
**Marcovecchio 2014** [81]	206 (107 M, 99 F) (11–18) yrs	Nd	MA+ vs. MA-: Differences in final H (cm) 4.29 cm, *p* = 0.001	ΔH SDS during puberty: All:−0.148, *p* < 0.001 MA+ −0.286 vs. MA- 0.083; *p* = 0.023	In the group as a whole, mean H SDS significantly declined during puberty, *p* < 0.001. In MA+ group the H SDS declined during puberty greater than MA- group (*p* = 0.023).	
**Plamper 2017** [82]	1294 (664 M, 630 F) (7–16) yrs	H SDS at study start (Mean SDS): All 0.17 M 0.17 F 0.17	H SDS at study end (Mean SDS): All 0.01 M −0.02 F 0.04		Mean HV at the peak of the growth spurt was significantly reduced in M (*p* < 0.001) but not in F. In F: HV declined more rapidly after reaching pubertal peak HV compared to the reference population.	For both M and F, H SDS during the study period differed significantly from the reference population (*p* < 0.0001). The growth impairment was more pronounced in patients with poor metabolic control.
**Luna 2005** [83]	83 (46 M, 37 F) (8 yrs—adult bone age)	M: 0.08 ± 0.16 F: 0.40 ± 0.16	M: −1.02 ± 0.27 F: 0.13 ± 0.24		Final H SDS was significantly decreased compared to H at diagnosis in M (*p* < 0.01). In F final H SDS was lower compared to H at diagnosis but not significantly (*p* < 0.05).	Apparently minor role of metabolic control .
**Kanumakala 2002** [84]	92 (43 M, 49 F) (8–18) yrs	M: −0.17 ± 0.99 F: −0.29 ± 1.19	M: −0.39 ± 0.99 F: −0.13 ± 1.07		Normal mean Final H compared to the general population.	No correlation between metabolic control and linear growth.
**Huang 2001** [85]	44 (317 M, 270 F) (0.3–20.3) yrs	M: −0.03 ± 0.67 F: 0.44 ± 0.91	M: −0.13 ± 0.66 F: −0.05 ± 0.86		Mean final H had a lower SDS than H at diagnosis (*p* = 0.009). Both M and F attained a normal mean final H compared to their GTH (*p* < 0.005).	The final H as well as the reduction in H SDS was not correlated with the age at diagnosis or with metabolic control.
**Parthasarathy 2016** [90]	160 (75 M, 85 F) (4–16) yrs	H SDS at study start (Mean SDS ± SD): 0.9 ± 1.3	Nd	HV at study end: (Mean SDS ± SD): −0.3 ± 1.5	T1DM children had lower HV than healthy children	Children on basal-bolus therapy had higher HV those on a split mix regimen (*p* < 0.05). Children diagnosed before 5 yr had lowest HV.
**Choudhury 2000** [91]	61 (39 M, 22 F) (3.7–16.8) yrs	All:−0.095 ± 0.96 M: −0.091 ± 0.95 F: −0.10 ± 0.93	Data on 12 children: −0.24 (±1.24)	H SDS in M with onset <5 yr: 0.39 (±0.75), *p* < 0.05	M with onset before 5 yrs were significantly taller than standard population. Normal prepubertal linear growth.	
**Demir 2010** [93]	101 (55 M, 46 F) (2–15.5) yrs	M: 0.3 ± 1.1 F: 0.1 ± 0.9	H SDS at study end: M: 0.1 ± 1 F: −0.3 ± 0.9		H at diagnosis, for both M and F, was significantly higher compared to GTH. Mean H SDS did not change significantly during the follow-up period.	Mean HbA1c showed a negative correlation with ΔH SDS at the 3rd year of diagnosis (*p* = 0.03).

F: Females, M: Male, H: height, HV: height velocity, GTH: Genetic target height, IGF-1: Insulin-like growth factor-1, IGFBP3: Insulin-like growth factor binding protein 3, SDS: Standard deviation score, HbA1c: Glycated haemoglobin, MA+: Microalbuminuric group, MA-: Normoalbuminuric group; *Data were compared to those of healthy controls matched for age and sex. ; ^Data on prepubertal growth.

## 5. The Effects of Insulin Therapy on Linear Growth in Patients with Type 1 Diabetes Mellitus (T1DM)

As in every chronic disease, ensuring normal growth during childhood and adolescence is one of the therapeutic targets of good management of T1DM. Subcutaneous long-acting insulin or the continuous subcutaneous infusion of rapid-acting insulin to cover basal requirements and with rapid-acting insulin to prevent or correct glucose excursions is considered the standard of care. In recent decades, new intensive insulin regimens based on multiple dose injection (MDI) or continuous subcutaneous insulin infusion (CSII), together with regular glucose monitoring, have considerably improved metabolic control in patients with T1DM, but their effects on linear growth are still uncertain according to the literature.

It was recently demonstrated that adult height SDS could be positively influenced by good basal insulinization during puberty [59]. On the other hand, the use of CSII therapy does not seem to improve chronic hepatic hypo-insulinization and does not seem to influence the linear growth pattern compared to MDI therapy [31].

The correlation between the different types of insulin regimens and linear growth has been investigated by many authors. Parthasarathy et al., in their recent study in 160 children with T1DM, reported that patients on basal-bolus therapy had better metabolic control and a higher growth velocity than the group on split mix insulin (HbA1c 8.4 ± 1.7% vs. 9.0 ± 1.8%; growth velocity 0.5 ± 1.6 vs. −0.3 ± 1.4, respectively) [90]. Ekstrom et al. demonstrated that the start of glargine insulin treatment in a small group of 12 adolescents led to a decreased nocturnal secretion of IGFBP-1, an increased level of IGF1, and an improvement of HbA1c compared to neutral protamine hagedorn (NPH) insulin. This effect could be due to the prolonged action of glargine in comparison with NPH insulin and to the consequent increase in nocturnal hepatic insulinization [95]. However, Cherubini et al., in a randomized crossover study involving 15 prepubertal children, did not find any significant differences in the GH–IGF-1 axis with the use of glargine or detemir insulin and found increased weight and height gains in children treated with detemir insulin [96].

## 6. Conclusions

Normal growth parameters are important indicators of good disease control in children and adolescents with T1DM. To date, although limited data are available, the literature has confirmed the presence of some growth anomalies in children and adolescents with T1DM. Many studies have shown an increased height at diagnosis of the disease, particularly in children with early onset, and most reports have demonstrated impaired linear growth at prepubertal and pubertal age. It has also been demonstrated that T1DM duration and metabolic control could influence children’s linear growth pattern and final adult height.

Though modern therapies based on MDI or CSII with new insulin analogues ensure more physiological insulin supplementation than previous therapies, some anomalies of the GH/IGF-1 axis are still detectable due to persistent hepatic hypo-insulinization. However, modern intensive insulin regimens, leading to improved metabolic control, are able to ensure a normal adult height in line with patients’ target parameters [22,59]. Therefore, currently, the choice of the most appropriate therapeutic regimen to achieve a good insulin level and the best metabolic control for each patient, together with the regular measurement of growth parameters, remains the most important available tool for a pediatric diabetologist.

Nevertheless, in recent years, many technological advances, such as sensor-augmented pumps and closed-loop systems, have been introduced in routine therapy for T1DM patients with the aim of improving metabolic control in patients with T1DM, and no data on growth parameters are available for these therapies. Since these new technologies are the therapy of choice in young children, especially those of pre-school age [97], it would be of great interest to evaluate their effects on the growth pattern of children with T1DM.
